# Inorganic–organic hybrid materials through post-synthesis modification: Impact of the treatment with azides on the mesopore structure

**DOI:** 10.3762/bjnano.2.52

**Published:** 2011-08-26

**Authors:** Miriam Keppeler, Jürgen Holzbock, Johanna Akbarzadeh, Herwig Peterlik, Nicola Hüsing

**Affiliations:** 1 Inorganic Chemistry I, Ulm University, Albert-Einstein Allee 11, D-89081 Ulm, Germany; 2 Faculty of Physics, University of Vienna, Strudlhofgasse 4, A-1090 Vienna, Austria,; 3 Materials Chemistry, Paris-Lodron University Salzburg, Hellbrunner Str. 34, A-5020 Salzburg, Austria

**Keywords:** inorganic–organic hybrid materials, mesoporous materials, nucleophilic substitution, silica, sol–gel chemistry

## Abstract

Hybrid, hierarchically organized, monolithic silica gels, comprising periodically arranged mesopores and a cellular macroscopic network, have been prepared through a co-condensation reaction of tetrakis(2-hydroxyethyl)orthosilicate with chloromethyl-trimethoxysilane or 3-(chloropropyl)-triethoxysilane. Subsequent conversion of the chloro groups into azido groups, by nucleophilic substitution with NaN_3_ in *N*,*N*-dimethylformamide, was conducted upon preservation of the monolithic structure. However, treatment with NaN_3_ had a strong influence on the structure in the mesoporous regime, with changes such as an increase of mesopore diameter, pore volume and lattice constants, as well as a concomitant decrease of the pore wall thickness, as confirmed by small angle X-ray scattering, transmission electron microscopy, and nitrogen sorption analysis. Similar effects were observed for unmodified silica gels by simple ageing in azide-containing media, whether a relatively small or a sterically demanding counter ion (Na^+^ or (H_3_C)_4_N^+^) was used. The structural modification did not seem to depend greatly on whether an organic aprotic solvent (*N*,*N*-dimethylformamide, 1,1,3,3-tetramethylurea, 1,3-dimethyl-2-imidazolidinone) or a protic solvent that can form hydrogen bonds, such as water, was used.

## Introduction

Inorganic–organic hybrid materials with tailored porosity on several length scales are of interest for a variety of applications, such as separation, adsorption, catalysis, energy storage, etc., due to the benefits arising from each pore size regime, e.g., rapid mass transport through macropores combined with selectivity provided by meso- or micropores. This is especially true for materials with uniform pore size distributions in the mesoporous (2–50 nm) and/or macroporous regime (>50 nm) [1–3].

A powerful tool in the preparation of stable, mesoscopically organized materials that are characterized by narrow mesopore size distributions, high specific surface areas and large pore volumes, is the application of cooperative self-assembly processes between supramolecular aggregates of organic molecules, oligomers or polymers and inorganic species such as alkoxysilanes for silica-based materials [4–12]. Since the first presentation by Kresge and Beck in 1992, these so-called M41S-materials have attracted great attention and their formation mechanism as well as the parameters influencing the textural properties have been well investigated [4–5]. It is well-known that the manipulation of the dimensions and the state of aggregation of the supramolecular aggregates will directly influence the structural properties of the resulting inorganic porous material, and research efforts are devoted to the control of the structural properties through the synthesis conditions. For ordered mesoporous materials, whose syntheses are based on block copolymers such ethylene oxide (EO)*_x_*–propylene oxide (PO)*_y_*–ethylene oxide (EO)*_x_*, this can be achieved by variation of the length of the EO or PO blocks, by increasing the synthesis temperature or by the addition of inorganic salts [6,9,13]. Currently, progress in the synthesis protocols even allows for the preparation of different macroscopic morphologies such as powders, coatings, fibres, or monoliths.

Monoliths are of special interest for functional devices, e.g., as chromatography columns, or catalytic reactors. However, to allow mass transport with a minimal pressure build-up the presence of a macroporous network is indispensable. Such a second level of porosity in mesoporous silica monoliths can be introduced by several synthetic approaches: Dual templating with sacrificial templates, phase separation processes (e.g., based on polymers), or the application of diol/polyol-modified silanes [1–3,14–18].

Nakanishi and Lindén relied on polymerization-induced phase separation during sol–gel processing to form monolithic bodies with a hierarchical organisation of the pore structure at the meso- and macroscopic length scale [16–17]. The materials obtained were characterized by interconnected porosity on several length scales. The macropore diameter was controlled through PEO–siloxane interactions, whereas the mesopore diameter was governed by the presence of the surfactant, e.g., cetyltrimethylammonium bromide or a poly(ethylene oxide)-based polymer. In our group, silicon diolates in the presence of surfactants were applied for the preparation of monolithic gels (silica and inorganic–organic hybrid networks) with a cellular network built up of macropores of about 2 μm diameter and periodically arranged mesopores of 7 nm [18]. Each of these strategies allows for a high level of control over macropore/mesopore size distribution, surface area, etc.

It is well known that ageing of silica gels in different environments or in hydrothermal conditions has a pronounced influence on the final gel structure [19–20]. Ageing at 100 °C in an autoclave yields mesoporous silica gels with larger pore sizes and pore volumes compared to gels aged in ethanol at room temperature due to promoted dissolution and reprecipitation processes [20]. Processes such as syneresis, Ostwald ripening, etc., are facilitated and accelerated with increasing temperature and pressure. The same is true for gels with periodically arranged mesopores or even mesostructured cellular foams [21].

Structural arrangements can be quite pronounced depending on the conditions of the post-treatment. One example is the so-called pseudomorphic transformation, in alkaline solutions and hydrothermal conditions, from amorphous mesoporous materials to well-organized mesoporous structures [22–23].

Many silica gels are functionalized by organic groups specific for their eventual applications. Typical examples are the hydrophobization with methyl or phenyl groups, and even functional groups such as polymerizable moieties or metal-coordinating groups can be introduced [24]. These groups are typically incorporated either by post-synthetic grafting processes or by co-condensation reactions of different alkoxysilanes. The impact of these synthesis steps on the final pore structure is quite well investigated [25]. In post-synthetic functionalization procedures, a porous matrix with the desired pore size, pore connectivity, surface area, etc., is prepared prior to the modification step and the organic moieties are made to react with the surface silanol groups in a second step. For this approach, it is assumed that structural changes are minimal [25]. For the co-condensation approach, in which tetraalkoxysilanes [Si(OR)_4_] are condensed to form an inorganic network in the presence of organically substituted tri-alkoxysilanes [R'–Si(OR)_3_], network formation and thus structural features such as pore size, connectivity, etc., are strongly influenced by the presence of the organosilane [25].

These organo-functionalized silica gels can be further modified by chemical reactions with more complex functional groups; recent examples include Cu(I)-catalyzed 1,3-dipolar cycloadditions, also termed Click reactions, on silica surfaces involving alkynes and azide functionalities [26–28]. Reviewing the literature on this topic reveals that most of the examples of postsynthesis surface chemical reactions are concerned with the successful chemical conversion, but the structure of the modified materials is in many cases not characterized in great detail. In a recent work, we have shown that prior to the Click reaction, the conversion from chloro to azido functionalities in silica monoliths is possible, but that this reaction concomitantly occurs with major structural changes [29].

The present work focuses on the influence of these surface functionalization reactions on the structural properties of preformed silica gels. The first section describes the nucleophilic substitution of hierarchically organized SiO_2_–(CH_2_)_1,3_–Cl gels to give the corresponding SiO_2_–(CH_2_)_1,3_–N_3_ gels in a saturated NaN_3_/DMF solution, with special focus on the structural changes of the silica backbone. In a second section the influence of different solvents and counter ions is discussed for unmodified hierarchically organized SiO_2_ gels as reference samples.

## Results and Discussion

### Nucleophilic substitution of chloro- by azido groups on the silica surface

**Figure 1:**
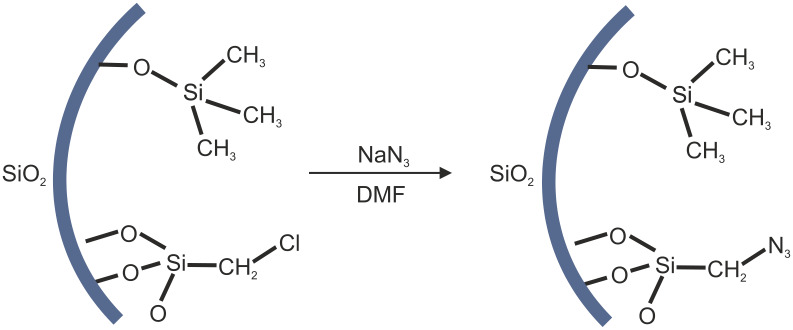
Schematic description of the nucleophilic substitution reaction for chloromethyl-modified silica pore surfaces.

Nucleophilic substitution of chloroalkyl-modified silica monoliths to azide-containing monoliths (SiO_2_–(CH_2_)_1,3_–Cl → SiO_2_–(CH_2_)_1,3_–N_3_) was conducted in a saturated solution of NaN_3_ in DMF on monolithic silica gels that had been treated with trimethylchlorosilane (Figure 1). During the course of this reaction, the macroscopic morphology of the monoliths was retained, and no significant influence on the macroporous network was observed. The gels had been treated with trimethylchlorosilane to remove reactive silanol groups and facilitate drying of the monoliths.

Gels modified with chloromethyl groups (CMTMS) or chloropropyl groups (CPES) were subjected to the azide solutions. The number of azide groups per nm^2^ was evaluated according to a previously published method and was found to be in the range of 0.7 nm^−2^ (3.0 mmol CMTMS), 1.2 nm^−2^ (4.5 mmol CMTMS), 1.3 nm^−2^ (6.0 mmol CMTMS) and 0.7 nm^−2^ (3.0 mmol CPES) at a reaction temperature of 60 °C [29]. The presence of the newly inserted azide functionalities was also confirmed by IR–ATR spectroscopy. This type of reaction has previously been reviewed for a variety of different silica surfaces, however, the influence of the reaction on the structural properties of the material has been mostly neglected [30].

**Figure 2:**
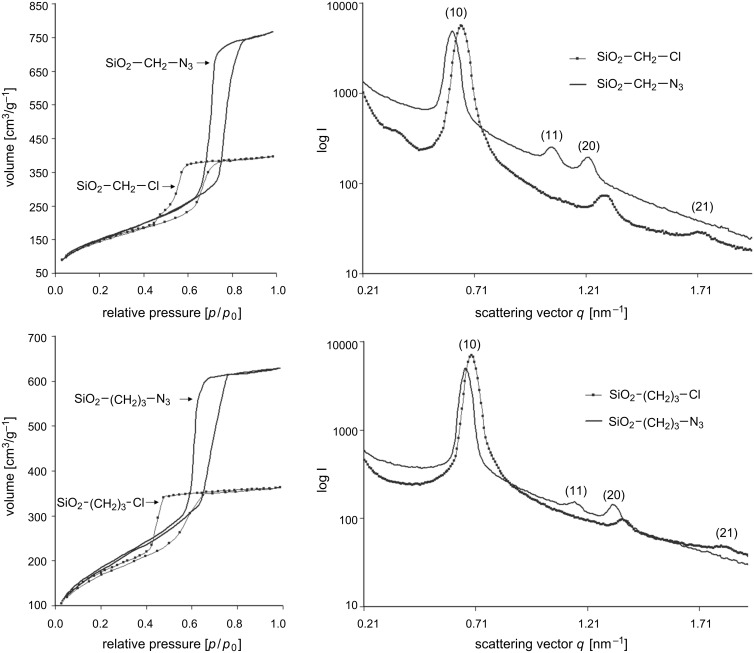
Nitrogen sorption isotherms (taken at 77 K, left) and SAXS patterns (right) of SiO_2_–(CH_2_)*_n_*–Cl and SiO_2_–(CH_2_)*_n_*–N_3_ gels after nucleophilic substitution in a saturated DMF/NaN_3_ solution at 60 °C. *n* = 1 (top): Prepared from a silica precursor solution containing 4.5 mmol CMTMS; *n* = 3 (bottom): Prepared from a silica precursor solution containing 3.0 mmol CPES.

This influence of the nucleophilic substitution on the porous structure of the meso/macroporous monoliths, with special emphasis on the long range hexagonal ordering of the mesopores, was evaluated by nitrogen sorption and small angle X-ray scattering (SAXS) analyses. Figure 2 shows the nitrogen adsorption/desorption isotherms at 77 K for modified silica gels before and after nucleophilic substitution (SiO_2_–(CH_2_)_1,3_–Cl → SiO_2_–(CH_2_)_1,3_–N_3_). The isotherms for the chloroalkyl-containing precursor materials are of type IV with H2 hysteresis loops according to the classification of Sing et al. [31], whereas the same samples after conversion of the chlorides into azides display hysteresis loops of H1 type indicating a narrow distribution of pores. In addition, the isotherms for SiO_2_–(CH_2_)_1,3_–N_3_ exhibit stretching along the volume axis, adsorption and desorption isotherms display a sharper capillary condensation step and the relative pressure of the pore filling is shifted to larger values compared to the corresponding SiO_2_–(CH_2_)_1,3_–Cl. These variations in the hysteresis loops indicate an increase in the pore diameter and still a narrow pore size distribution for SiO_2_–(CH_2_)_1,3_–N_3_.

The H2 type hysteresis loops obtained for SiO_2_–(CH_2_)_1,3_–Cl suggest rather complex pore structures with interconnected pores of different size and shape, e.g., spherical mesopores interconnected by smaller windows or large pore channels with undulating walls are possible. The pore sizes calculated from the desorption branch of the isotherm, applying the Barrett–Joyner–Halenda (BJH) model, are in the range of 3.5–4.7 nm for all samples. However, for pore diameters smaller than 5 nm (in our case presumably given by the small interconnecting windows; see Table 1, *D*_BJH,Des_) the relative pressure at which desorption occurs is strongly influenced by fluid cavitations and instability of the meniscus [32]. In addition, the BJH model is based on the Kelvin equation, which describes the relationship between the relative vapour pressure in equilibrium and the radius of curvature of the meniscus [33]. Since a stable fluid meniscus with a given radius of curvature cannot be guaranteed for the desorption process in all systems, and the risk of obtaining physically meaningless results exists, the adsorption branch was also used to calculate the pore size distribution. This is not the case for the azido-functionalized samples (SiO_2_–(CH_2_)_1,3_–N_3_) with pore sizes larger than 5.4 nm for all samples. Here, the calculation using the desorption isotherm is favoured, since desorption processes are thermodynamically more stable compared to the corresponding adsorption processes.

**Table 1:** Structural characteristics of SiO_2_–(CH_2_)_1,3_–Cl compared to corresponding SiO_2_–(CH_2_)_1,3_–N_3_, obtained from nitrogen sorption analysis at 77 K.

	*S*_BET_^a^ [m^2^ g^−1^]		*C*_BET_		*V*_max_ [cm^3^ g^−1^]		*D*_BJH,Des_ [nm]^b^		*D*_BJH,Ads_ [nm]^c^	

	SiO_2_–Cl	SiO_2_–N_3_	SiO_2_–Cl	SiO_2_–N_3_	SiO_2_–Cl	SiO_2_–N_3_	SiO_2_–Cl	SiO_2_–N_3_	SiO_2_–Cl	SiO_2_–N_3_

3.0 mmol CMTMS	566	513	80.0	56.7	347.5	650.2	3.69	6.74	5.54	9.17
4.5 mmol CMTMS	529	556	64.7	66.7	397.2	768.2	4.68	7.34	6.36	9.12
6.0 mmol CMTMS	445	563	56.3	55.4	325.7	768.5	3.70	7.33	5.48	9.13
3.0 mmol CPES	611	664	74.8	55.7	363.0	630.0	3.50	5.37	4.82	6.34

^a^Calculated by using the Brunauer–Emmett–Teller (BET) model. ^b^Calculated from the desorption isotherm by using the BJH model. ^c^Calculated from the adsorption isotherm by using the BJH model.

Table 1 gives all pore sizes as calculated from the adsorption and desorption isotherms. As expected, the calculation from the adsorption isotherm led to larger pore diameters for all samples with differences in the desorption pore size in the range of 1.7 to 2.4 nm for the methyl-spacer samples (*n* = 1), and in the range of 1.0 to 1.3 nm for the propyl-spacer samples (*n* = 3). Regardless of which sorption branch was applied for the calculation, a significant enlargement in the mesopore diameter after nucleophilic substitution in the range of 2.7 to 3.7 nm for methyl-spacer samples and in the range of 1.5 to 1.9 nm for propyl-spacer samples was observed. For instance, the chloromethyl-modified sample (3.0 mmol CMTMS) showed a pore diameter *D*_BJH,Ads_ of 5.54 nm prior to nucleophilic substitution and after conversion into the azides an increase to *D*_BJH,Ads_ = 9.17 nm was detected. The larger amount of nitrogen adsorbed at relative pressures above *p*/*p*_0_ = 0.3 indicates a dramatic increase of the specific pore volumes (*V*_max_ and *V*_meso_) after nucleophilic substitution, but relatively constant specific surface areas were observed from the pressure range *p*/*p*_0_ = 0.05–0.30). *V*_max_ and *V*_meso_ followed the same trend and showed only slight deviations in their values, thus only *V*_max_ is discussed in the course of this work. The difference in *V*_max_ between samples before and after nucleophilic substitution was in the range of 300 to 440 cm^3^ g^−1^ for methyl-spacer samples and in the range of 270 cm^3^ g^−1^ for propyl-spacer samples (Table 1).

The decreasing *C*-value, indicative of the adsorbent–adsorbate interactions, for gels prepared from a silica-precursor solution containing 3.0–6.0 mmol CMTMS follows the trend expected for gels with increasing coverage of the silica surface with organic groups. For nitrogen sorption on non-modified silica materials, the *C*_BET_ values are typically in the range 80–150 [34].

Figure 2 also shows the SAXS patterns for the modified silica gels before and after nucleophilic substitution of the chlorides into azides (SiO_2_–(CH_2_)_1,3_–Cl → SiO_2_–(CH_2_)_1,3_–N_3_). For all samples, higher order reflections were found, indicating long range ordering of the pore system. SiO_2_–(CH_2_)_1,3_–N_3_ exhibited the characteristic Bragg reflection sequence for a 2-D hexagonal ordering of 1 : 3^1/2^ : 2 : 7^1/2^… and the reflections were indexed to the (10)-, (11)- and (20)-crystallographic planes [35]. A comparison with SAXS patterns of the corresponding SiO_2_–(CH_2_)_1,3_–Cl precursor material clearly indicates mesostructural changes during the process of nucleophilic substitution. Both, SiO_2_–(CH_2_)_1,3_–Cl as well as SiO_2_–(CH_2_)_1,3_–N_3_ showed typical diffraction patterns for a 2-D hexagonal ordering of the pores. However, the relative intensities of the reflections were different for the chloroalkyl-modified silica gels compared to the corresponding azido-modified gels. The intensity of the (11)-reflection was reduced (almost to zero) compared to the corresponding azidoalkyl-modified silica gels. Furthermore, an additional higher order reflection was found for the chloroalkyl-modified precursor material that can be indexed to the (21)-crystallographic plane.

The differences in the reflection intensities before and after nucleophilic substitution (Figure 2) are attributed to the different form factors arising from differences in the respective pore wall thicknesses and pore diameters. One approach to describe these intensities is a two-phase model (pore and silica), where the form factor can be analytically solved (for more details see Supporting Information File 1 and [36]). This model has been previously used to determine the pore diameter and pore-wall thickness of surface functionalized silica gel monoliths [37].

Another approach is based on the reconstruction of the electron densities from a Fourier series and the appropriate choice of the phases [38–39]. This has been experimentally and theoretically used to model the electron density across the pore for modified and unmodified MCM-41 and SBA-15 materials [11,40] (for detailed information on SAXS data evaluation, see Supporting Information File 1).

The best solution for the three observed reflections in our case was −+−, which differs to the phase shift from −−++ for the first four coefficients for the SBA 15 material observed by Flodström et al. or −++− for the MCM 41 material [11,40]. This could be due to the variations in the synthesis conditions of the different materials.

**Figure 3:**
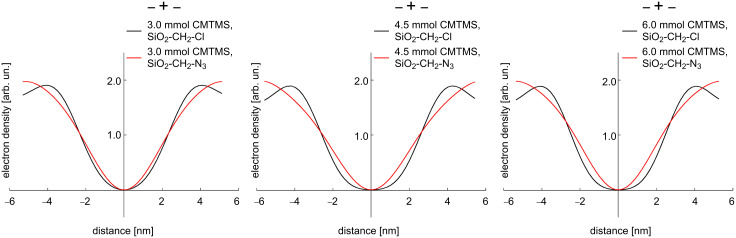
Electron density reconstructions for modified silica gels (SiO_2_–CH_2_–Cl and SiO_2_–CH_2_–N_3_) that have been prepared from a silica precursor solution containing 3.0 (left), 4.5 (middle) and 6.0 (right) mmol CMTMS. The electron density of SiO_2_–CH_2_–Cl corresponds to a sharper interface, whereas the broader distribution of SiO_2_–CH_2_–N_3_ indicates a higher surface roughness due to the nucleophilic substitution.

As an example, in Figure 3, the electron density reconstructions are shown for the SiO_2_–CH_2_–Cl and SiO_2_–CH_2_–N_3_ gels, with SiO_2_–CH_2_–Cl exhibiting a smaller pore with a steeper slope of the electron densities, whereas the corresponding substituted gel (SiO_2_–CH_2_–N_3_) has a broader distribution, which indicates a larger pore with a higher surface roughness.

One would expect the electron density to converge to a constant value within the silica phase. Unfortunately, due to the limited number of peaks available for the reconstruction, the resolution was limited [38–39]. A large constant region would require the sum of a large number of Fourier coefficients, i.e., a large number of diffraction peaks, which are not available for our type of materials. Thus this is an inevitable inherent weakness of the model.

The change in the ratio of the silica wall thickness to the pore diameter, during the nucleophilic substitution process, was also evidenced by nitrogen sorption analysis. An increase in pore diameter was observed (Table 1), while simultaneously a reduction of the pore wall thickness was detected for SiO_2_–(CH_2_)_1,3_–N_3_ compared to SiO_2_–(CH_2_)_1,3_–Cl (Table 2).

**Table 2:** Comparison of the structural characteristics of SiO_2_−(CH_2_)_1,3_−Cl and the corresponding SiO_2_−(CH_2_)_1,3_−N_3_.

		mean pore diameter ± 0.3^a^ [nm]		mean wall thickness ± 0.3^a^ [nm]		*t*_Des_^b^ [nm]		*t*_Ads_^c^ [nm]	

		SiO_2_–Cl	SiO_2_–N_3_	SiO_2_–Cl	SiO_2_–N_3_	SiO_2_–Cl	SiO_2_–N_3_	SiO_2_–Cl	SiO_2_–N_3_

3.0 mmol CMTMS		6.60	7.85	4.00	3.15	6.92	4.22	5.07	1.79
4.5 mmol CMTMS		7.05	8.75	4.15	3.25	6.52	4.51	4.84	2.73
6.0 mmol CMTMS		6.65	8.10	4.05	3.15	7.02	3.87	5.24	2.07
3.0 mmol CPES		6.50	7.70	4.00	3.30	7.00	5.59	5.68	4.62

^a^Mean pore diameter and wall thickness calculated from the peak intensities using a two-phase model with an analytical approach. ^b^Wall thickness, calculated by: Lattice constant *a* − *D*_BJH,Des_. ^c^Wall thickness, calculated by: Lattice parameter *a* − *D*_BJH,Ads._

**Figure 4:**
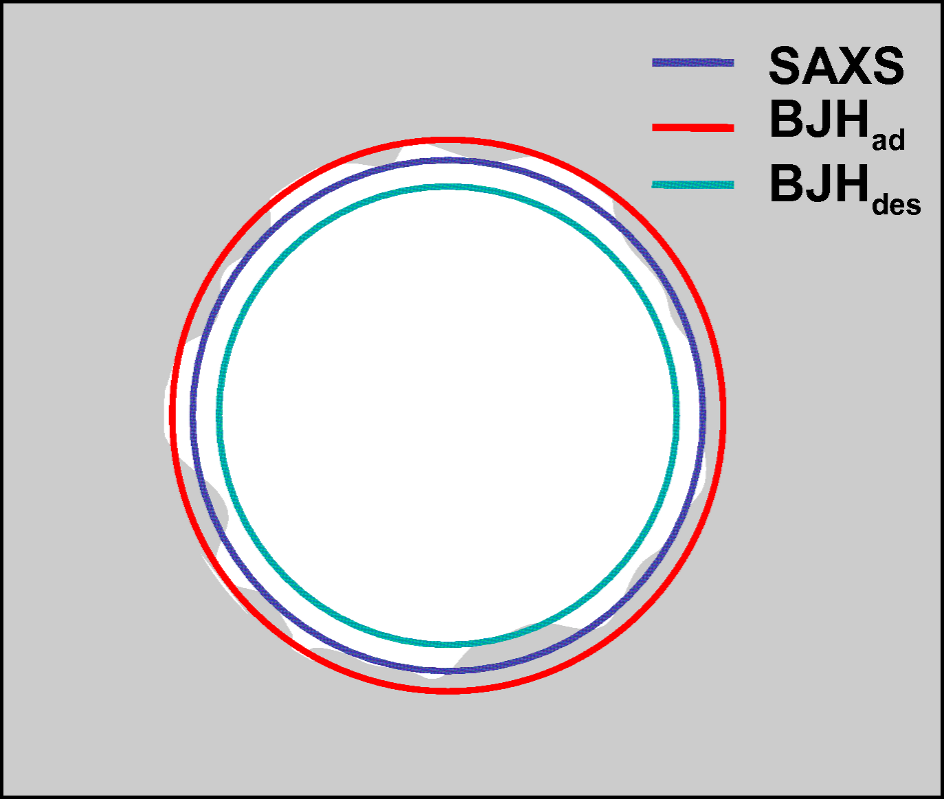
SAXS averages the surface inhomogeneities to a mean radius in the two-phase model and leads therefore to a slightly larger diameter than the nitrogen sorption analysis (desorption branch).

In the SAXS experiments, this led to the striking appearance of the (11)-reflection and the disappearance of the (21)-reflection. However, whereas sorption analysis indicated a strong decrease of the pore wall thickness, this effect was much less pronounced for the SAXS measurements. One possible explanation could be an additional surface roughness of the pores, which is also in coincidence with the electron density reconstruction (Figure 3). The model description in SAXS (see Supporting Information File 1) as a two-phase material, i.e., cylindrical pores of identical radius embedded in a silica matrix, leads to the measurement of a mean radius and averages out any differences in the radii or effects from surface inhomogeneities or roughness along the length or cross section of the pore. The radius obtained from SAXS could then lie intermediate between the radius obtained in the BJH analysis, from the adsorption branch and that from the desorption branch, as shown in Figure 4. In the desorption branch, the BJH analysis is restricted, by the presence of small pores or surface roughness, to give the smallest pore size (Figure 4).

Not only did the reflection intensity change during nucleophilic substitution, but also the relative position of the scattering vector q_(hk)_ shifted to smaller values (Table 3), indicating an increase of the repeating unit distance. For instance, the chloromethyl-modified sample (4.5 mmol CMTMS) showed a shift of the scattering vector *q*_(10)_ from 0.65 to 0.61 nm^−1^ after conversion of the chlorides into the azides, corresponding to an increase of the *d*_(10)_-spacing from 9.66 nm to 10.26 nm. The *d*_(10)_-spacing was used to calculated the lattice constant, which was also found to increase during nucleophilic substitution (Table 3). For example, the chloromethyl-modified sample showed an increase in the lattice constant from 11.15 nm to 11.85 nm during nucleophilic substitution.

**Table 3:** Structural properties of SiO_2_–(CH_2_)_1,3_–Cl compared to corresponding SiO_2_–(CH_2_)_1,3_–N_3_ obtained from SAXS analysis.

		*q*_(10)_^a^ [nm^−1^]		*d*_(10)_^a^ [nm]		*a*^b^ [nm]	

		SiO_2_–Cl	SiO_2_–N_3_	SiO_2_–Cl	SiO_2_–N_3_	SiO_2_–Cl	SiO_2_–N_3_

3.0 mmol CMTMS		0.69	0.66	9.17	9.51	10.58	10.98
4.5 mmol CMTMS		0.65	0.61	9.66	10.26	11.15	11.85
6.0 mmol CMTMS		0.68	0.65	9.26	9.72	10.69	11.23
3.0 mmol CPES		0.69	0.67	9.10	9.45	10.50	10.91

^a^Calculated from SAXS measurements, *q*_(10)_ = 4π/λ·sinΘ, *d*_(10)_ calculated by the Bragg equation. ^b^Lattice constant, calculated by 2*d*_(10)_/(3)^1/2^.

**Figure 5:**
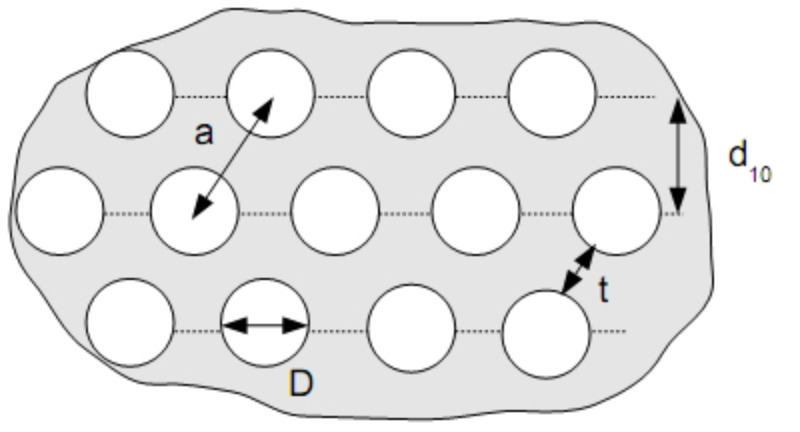
Schematic representation of a hexagonally organized pore system with the characteristic sizes. A similar arrangement is found for the samples by SAXS and transmission electron microscopy (TEM) analyses.

The structural parameters obtained from nitrogen sorption and SAXS analyses suggest a process of mesostructural changes during conversion of SiO_2_–(CH_2_)_1,3_–Cl into the corresponding SiO_2_–(CH_2_)_1,3_–N_3_. Figure 5 shows schematically a hexagonally organized porous material with cell parameters *a*, wall thickness *t*, repeating unit distance *d*_10_ and pore diameter *D* as obtained from nitrogen sorption.

Based on the nitrogen sorption and SAXS analysis, the following trends were observed during nucleophilic substitution: The mesopore diameter and maximal pore volume drastically increased, while the lattice constant showed only a small enlargement with a simultaneous decrease in pore wall thickness. The ratio of pore wall thickness to pore diameter decreased to such an extent, that the new electron density (phase shift of Fourier coefficients) involved a significant change in the reflection intensity. We assume that the reduction in pore wall thickness with a simultaneous increase in pore diameter can not simply be explained by dissolution processes of silica, because the lattice constants also increased during the nucleophilic substitution. Simple ageing of unmodified mesoscopically organized silica gels in azide-containing media allows us to demonstrate that the observed effects are not due to the inserted azide-functionalities, which are covalently attached to the silica surface, but rather to an exposure of a mesostructured silica matrix to azide ions, as presented vide infra.

### Ageing of unmodified silica gels in azide-containing media

**Figure 6:**
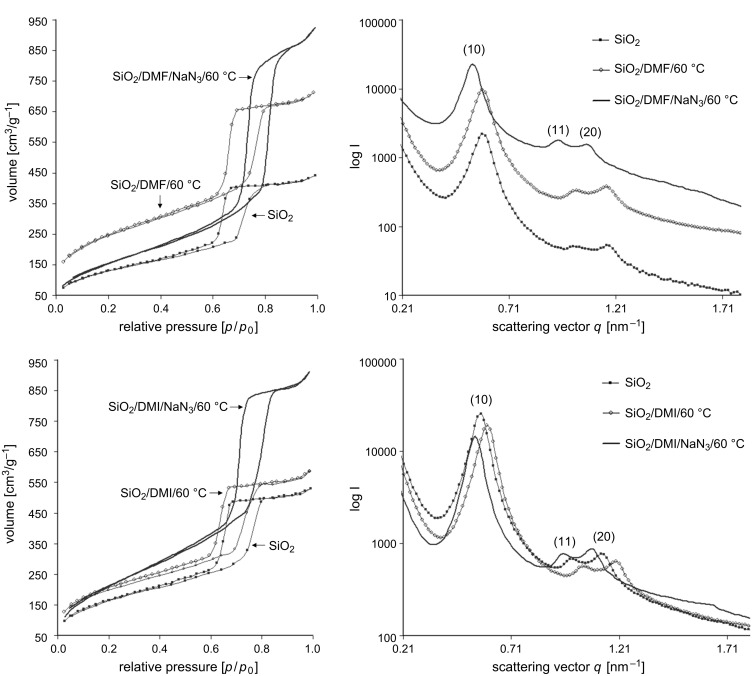
Nitrogen isotherms and SAXS patterns of untreated silica gels, reference silica gels (solvent/60 °C) and azide-treated silica gels (solvent/NaN_3_/60 °C) in different solvents: DMF (top) and DMI (bottom).

From the results obtained above for the chloroalkyl-modified silica gels, the cause of the structural changes cannot be identified clearly and without ambiguity. Therefore, the reaction conditions were changed step by step to isolate the influence of temperature, solvent, anion-cation pair and solvent-azide compositions to identify the critical parameter. Pure (not organically modified) silica gels were kept for 3 d at 60 °C (identical conditions as for the nucleophilic substitution described above) in solutions of NaN_3_ in different solvents ranging from *N*,*N*-dimethylformamide (DMF), 1,1,3,3-tetramethylurea (TMU), 1,3-dimethyl-2-imidazolidinone (DMI) to H_2_O. Reference samples were kept for 3 d at 60 °C in the respective solvents without azide and pure silica gels were aged at that temperature. In addition, sodium azide was changed to tetramethylammoniumazide ((H_3_C)_4_NN_3_, TMAA). All gels were aged, treated with trimethylchlorosilane and dried. The structural characteristics of untreated, reference and silica gels that were exposed to the different reaction conditions were again determined by nitrogen sorption and SAXS analyses. Note that the untreated silica, reference silica and azide-treated silica gels originated from the same monolithic silica piece, which was divided into three parts. Figure 6 shows the nitrogen adsorption/desorption isotherms taken at 77 K for gels treated in DMF and DMI; detailed information on gels in H_2_O and TMU is given in Supporting Information File 1.

All isotherms are of type IV with H1 hysteresis loops according to the classification of Sing et al. [33]. The reference samples that were heat treated in the various solvents showed higher pore volumes compared to untreated silica gels, but this effect was clearly intensified by the addition of NaN_3_, as demonstrated by the stretching of the hysteresis loops along the volume axis. Furthermore, the addition of NaN_3_ led to a shift of the relative pressure of the pore filling, by capillary condensation, to higher values. Pore diameters were significantly increased by the treatment with azide-containing solvents (Table 4). Differences in pore diameters for the various samples calculated from the adsorption isotherm in the BJH model were 4.05 nm (DMF/NaN_3_); 4.21 nm (TMU/NaN_3_); 2.20 nm (DMI/NaN_3_) and 2.25 nm (H_2_O/NaN_3_). The analogous calculation from the desorption branch led to smaller, but still significant, values for the increase in the pore diameter, i.e., 2.26 nm (DMF/NaN_3_); 1.77 nm (TMU/NaN_3_); 1.13 nm (DMI/NaN_3_) and 1.84 nm (H_2_O/NaN_3_).

**Table 4:** Structural characteristics of untreated silica, reference silica (solvent/60 °C) and azide-treated silica gels (solvent/NaN_3_/60 °C) from nitrogen sorption analysis at 77 K, solvents: DMF and DMI; azide: NaN_3_ and TMAA.

	*S_BET_*^a^ [m^2^ g^−1^]	*C*_BET_	*V*_max_ [cm^3^ g^−1^]	*D*_BJH,Des_^b^ [nm]	*D*_BJH,Ads_^c^ [nm]

SiO_2_	477	62.6	441.9	5.75	7.51
SiO_2_/DMF	876	103.3	712.7	6.27	9.24
SiO_2_/DMF/NaN_3_	592	42.2	924.8	8.01	11.56

SiO_2_	607	54.1	529.6	6.21	9.35
SiO_2_/ DMI	712	110.6	586.1	5.73	7.54
SiO_2_/DMI/NaN_3_	803	47.6	913.0	7.34	11.55

SiO_2_	627	62.0	554.4	6.23	9.21
SiO_2_/DMF	906	93.0	759.4	6.74	9.18
SiO_2_/DMF/TMAA	657	50.2	856.6	7.33	11.63

^a^Calculated in the BET model. ^b^Calculated from the desorption isotherm in the BJH model. ^c^Calculated from the adsorption isotherm in the BJH model.

Interestingly, the specific surface area *S*_BET_ dramatically increased from 477 m^2^ g^−1^ to 876 m^2^ g^−1^ by treatment of silica gels with pure DMF, whereas by treatment with DMF/NaN_3_ the *S*_BET_ value only slightly increased from 477 m^2^ g^−1^ to 592 m^2^ g^−1^. For the series with DMF this behaviour was reproduced for several samples (Table 4). The sample series with TMU and H_2_O showed the same behaviour, whereas for the series with DMI the sample with additional NaN_3_ exhibited the highest surface area (Table 4, and Supporting Information File 1).

Higher order reflections were found in the SAXS patterns for every sample, with the characteristic sequence for a 2-D hexagonal ordering of 1 : 3^1/2^ : 2 : 7^1/2^… [35]. As noted before for the series with SiO_2_–(CH_2_)_1,3_–Cl and SiO_2_–(CH_2_)_1,3_–N_3_, a variation in ratio of the radius of the high electron density region (that is the silica wall) to the inner pore volume was indicated by changes in relative reflection intensities. However, for the unmodified silica gels, neither untreated silica, reference silica nor azide-treated silica displayed the (21)-reflection or disappearance of the (11)-reflection as was seen for SiO_2_–(CH_2_)_1,3_–Cl or SiO_2_–(CH_2_)_1,3_–N_3._ This is also reflected in the electron density reconstruction (Figure 2, and Supporting Information File 1). We assume that this is due to differences in the electron density and pore wall thicknesses for unmodified silica compared to silica modified with organic functionalities covalently attached to the silica walls.

After treatment with solvent/NaN_3_ at 60 °C the relative positions of the scattering vectors *q*_(hk)_ shifted to smaller values (Table 5, and Supporting Information File 1) indicating an increase of the repeating unit distances. This was accompanied by an increase in the lattice constants, with a = 13.49 nm for samples that have been treated in DMF with the addition of NaN_3_, and a = 13.44 nm for the respective TMU and a = 13.39 nm for DMI samples. With H_2_O/NaN_3_ a slightly smaller lattice constant of 13.22 nm was observed (Supporting Information File 1).

**Table 5:** Structural properties as obtained from SAXS analysis of untreated silica, reference silica (solvent/60 °C) and azide-treated silica gels (solvent/NaN_3_ or TMAA/60 °C), solvents: DMF and DMI.

	*q*_(10)_^a^ [nm^−1^]	*d*_(10)_^a^ [nm]	*a*^b^ [nm]	*t*_Des_^c^ [nm]	*t*_Ads_^d^ [nm]	mean pore diameter^e^ ± 0.2 [nm]	mean wall thickness^e^ ± 0.2 [nm]

SiO_2_	0.59	10.73	12.39	6.68	4.92	8.60	3.80
SiO_2_/DMF	0.58	10.77	12.44	6.16	3.19	8.52	3.98
SiO_2_/DMF/NaN_3_	0.54	11.68	13.49	5.40	1.85	10.05	3.40

SiO_2_	0.56	11.13	12.85	6.53	3.39	8.60	4.20
SiO_2_/ DMI	0.60	10.55	12.18	6.40	4.59	8.15	4.05
SiO_2_/DMI/NaN_3_	0.54	11.60	13.39	6.07	1.86	9.20	4.20

SiO_2_	0.57	11.04	12.75	6.52	3.54	8.70	4.10
SiO_2_/DMF	0.61	10.27	11.86	5.12	2.68	8.15	3.85
SiO_2_/DMF/TMAA	0.55	11.33	13.08	5.75	1.45	9.75	3.55

^a^Calculated from SAXS measurements, *q*_(10)_ = (4π/λ)sinΘ, *d*_(10)_ calculated by the Bragg equation. ^b^Lattice constant, calculated by 2*d*_(10)_/(3)^1/2^. ^c^Wall thickness, calculated by: Lattice parameter *a* − *D*_BJH,Des_. ^d^Wall thickness, calculated by: Lattice parameter *a* − *D*_BJH,Ads_. ^e^Mean pore diameter and wall thickness calculated from the peak intensities (SAXS) using a two-phase model with an analytical approach.

With DMF, TMU and DMI we deliberately chose aprotic solvents that would not solvate the azide ions. This is important when azides are made to react by nucleophilic substitution, since assuming a bimolecular mechanism (S_N_2), the rate constant will be increased by a unsolvated, and therefore not stabilized, nucleophilic agent. This is in agreement with the fact that when methanol was used as the solvent for the nucleophilic substitution, the yield was much lower. However, the results from the series of gels treated in H_2_O clearly demonstrate that the effect on the mesostructure is due to the azide ions, independent of the coordination environment of the azide.

Substitution of NaN_3_ by (H_3_C)_4_NN_3_ (tetramethylammoniumazide, TMAA) led to similar effects on the mesostructure as mentioned above. An unmodified silica gel was kept for 3 d at 60 °C in a solution of TMAA in DMF. A reference sample was kept for 3 d at 60 °C in DMF. The structural characteristics of untreated silica, reference silica and silica gels that were exposed to DMF/TMAA (all originating from the same gel monolith) were again determined by nitrogen sorption and SAXS analyses.

**Figure 7:**
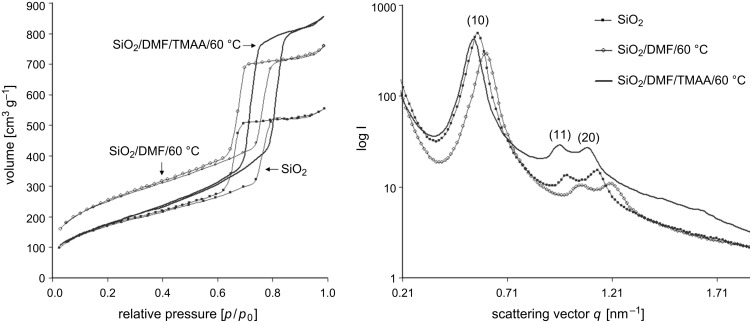
Nitrogen isotherms at 77 K and SAXS patterns of untreated silica, reference silica (DMF/60 °C) and azide-treated silica gels (DMF/TMAA/60 °C).

Figure 7 shows the nitrogen sorption isotherms and SAXS patterns. As observed previously for NaN_3_, the addition of TMAA leads to a shift of the relative pressure of the capillary condensation step to larger values, indicating an increase in mesopore diameter. Calculation from the desorption isotherm in the BJH model indicated an increase in the pore diameter from 6.23 to 7.33 nm, and calculation from the adsorption isotherm indicated an increase from 9.21 nm to 11.63 nm (Table 4). The specific surface area *S*_BET_ showed the same behaviour as for the series with DMF/NaN_3_ (Table 4). By treatment with pure DMF, a dramatic increase from 627 m^2^ g^−1^ to 906 m^2^ g^−1^ was observed, whereas by addition of the azide the *S*_BET_ value remained almost constant.

Higher order reflections with the same characteristic sequence for a 2-D hexagonal ordering of 1 : 3^1/2^ : 2 : 7^1/2^… were found. As observed for the series before, exposure to the azide compound led to a shift of positions for the scattering vectors *q*_(hk)_ to smaller values, indicating an increase of the repeating unit distance. In addition to that, an increase in the lattice constant was detected. A value of 13.08 nm was found for the sample that was treated with DMF and addition of TMAA in comparison to 12.75 nm for the untreated sample.

Exposure of silica gels to TMAA led to the same mesostructural effects as observed for NaN_3_. Therefore, substitution of a relatively small counter ion (Na^+^) by a sterically demanding counter ion ((H_3_C)_4_N^+^) did not change the observed effects of azides on mesoscopically organized silica gels.

## Conclusion

In summary, it was shown that a simple nucleophilic substitution reaction of chloroalkyl-functionalities on a silica surface to azidoalkyl-functionalities had an unexpected and drastic effect on the mesoporous structure. For such co-condensed silica samples with chloroalkyl-functionalities on the surface, an increase in the mesopore diameter, pore volumes (*V*_max_ and *V*_meso_) and lattice constant with a simultaneous decrease in pore wall thickness was observed upon nucleophilic substitution in NaN_3_ and DMF. Interestingly no influence on the macroscopic morphology (monoliths) and macroporous network was observed. In principle the same structural changes, albeit less pronounced, were observed for pure silica gels that have been treated in the presence of azide ions.

Further studies have shown that the structural changes can be related to the presence of the azide ions and are not due to the higher processing temperatures, counter ions or solvent molecules, as has been tested for *N*,*N*-dimethylformamide, 1,1,3,3-tetramethylurea, 1,3-dimethyl-2-imidazolidinone and water as the solvent, as well as tetramethylammonium cations as the counter ion.

Therefore, exposure to an azide-containing medium can be seen as a new postsynthetic approach to influence the mesostructural properties of highly porous silica gels.

## Experimental

**Materials**: Tetraethylorthosilicate (TEOS, Fluka), 3-(chloropropyl)-triethoxysilane (CPES, Aldrich), chloromethyl-trimethoxysilane (CMTMS, Wacker Chemie AG), and trimethylchlorosilane (TMCS, Merck) were used without further purification. Ethylene glycol (EG, Aldrich) was purified by drying with Na_2_SO_4_ and distillation from Mg. Pluronic P123 (*M*_av_ = 5800), EO_20_PO_70_EO_20_ (BASF) was applied without purification. For preparation of saturated azide solutions, sodium azide (Merck), tetramethylammoniumchloride (TMAC, Alfa Aesar) and tetramethylammoniumazide (TMAA, synthesized as described below), *N*,*N*-dimethylformamide (DMF, VWR), 1,3-dimethyl-2-imidazolidinone (DMI, Aldrich) and 1,1,3,3-tetramethylurea (TMU, Aldrich), were used without further purification.

**Preparation of hierarchically organized silica gels:** Tetrakis(2-hydroxyethyl)orthosilicate (EGMS) was synthesized according to Brandhuber et al. [41–42]. Hierarchically organized silica gels were prepared by condensation of EGMS in an aqueous reaction mixture containing P123 as the structure-directing agent and hydrochloric acid as the catalyst, according to a percentage by weight ratio of SiO_2_/P123/1M HCl : 18/30/70 wt %. The reaction mixture was homogenized for 1 min using a vortex stirrer to obtain a viscous white mixture, which was allowed to gel in a closed PP cylinder at 40 °C. The gels were kept at the same temperature for 7 d after gelation for ageing. Immersion of the wet gels in a solution of 10 wt % of trimethylchlorosilane (TMCS) in petroleum ether (PE) for 24 h and washing with PE and ethanol according to [37] resulted in complete expulsion of Pluronic P123, water and glycol.

**Preparation of chloroalkyl-modified silica gels**: Chloroalkyl-modified silica gels were prepared according to [29] by a co-condensation of EGMS and CMTMS or CPES in various molar ratios of EGMS:CMTMS = 9:1; 6.75:1 and 4.5:1. EGMS and CPES were used in a molar ratio of 9:1. The exact compositions can be found in Table 6. The gels were kept at the same temperature for 7 d after gelation for ageing. The wet gels were immersed in a solution of 10 wt % of trimethylchlorosilane (TMCS) in petroleum ether (PE) for 24 h to react with the free silanol groups, and washed by repeated immersion and storage of the whole monoliths into PE (three times within 24 h) and ethanol (five times within 48 h).

**Table 6:** Starting composition for chloroalkyl-modified mesostructured silica gels.

EGMS		chloroalkyltrialkoxysilane			template	

SiO_2_ content [%]^a^	amount [g]	organo-functional silane	amount [mL]	amount [mmol]	P123 [g]	1M HCl [g]

21.8	8.20	—	—	—	3	7
21.8	7.38	CMTMS	0.38	3.0	3	7
21.3	7.10	CMTMS	0.57	4.5	3	7
21.3	6.82	CMTMS	0.78	6.0	3	7
21.8	7.35	CPES	0.72	3.0	3	7

^a^Determined by TG analysis.

**Nucleophilic substitution:** The wet chloroalkyl-modified silica gels were immersed into a saturated solution of NaN_3_ in DMF at 60 °C, kept for 3 d and subsequently purified by repeated immersion in water (five times within 24 h) and ethanol (three times within 48 h) to remove unchanged NaN_3_. Drying of the wet silica gels was performed by simple evaporation of the solvent under reduced pressure at 60 °C.

**Ageing of the silica gels in azide-containing media:** Azide solutions of NaN_3_ in DMF or TMU were prepared by heating under reflux for 8 h at 80 °C followed by decantation from residual sediment at room temperature. A solution of NaN_3_ (0.1 g) in DMI (40 mL) was refluxed for 8 h at 80 °C, resulting in a transparent solution. A solution of NaN_3_ (0.1 g ) in H_2_O (40 mL) was prepared at room temperature, resulting in a transparent solution. A solution of TMAA in DMF was prepared by mixing 0.78 g TMAC (0.78 g) and NaN_3_ (0.46 g) in DMF (70 mL) followed by refluxing for 8 h at 80 °C and filtration. The wet silica gels were immersed into the azide solutions at 60 °C and kept for 3 d. Purification and drying of the wet gels was performed as describe above.

**Characterization:** The azide functionalities were detected by ATR–FT-IR spectroscopy using a Bruker Tensor 27. The density of the azides on the surface was calculated by the specific surface area (*S*_BET_) and the percentage of nitrogen, which was determined by elemental analysis of nitrogen using an ELEMENTAR Varino, according to:


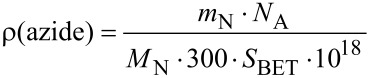


where *m*_N_ is the mass of nitrogen in 100 g of the silica gel, *N*_A_ is Avogadro’s constant, *M*_N_ is the molar mass of nitrogen and *S*_BET_ is the specific surface area according to the Brunauer–Emmett–Teller (BET) model.

Adsorption/desorption isotherms of nitrogen at 77 K were obtained with a NOVA 4000e (Quantachrome). Prior to analysis the samples were degassed at 60 °C for 3 h. The specific surface area was evaluated using sorption data in a relative pressure range of 0.05–0.30 with a five-point-analysis according to the BET model. Small angle X-ray scattering (SAXS) experiments were performed either with a Bruker Nano-Star or a Bruker Nano-Star (turbospeed solution) and a 2-D position sensitive detector (HiStar or Vantec 2000). Both instruments are equipped with a pinhole generator, where the X-ray beam is collimated and monochromatized by crossed Göbel mirrors.

The pore-to-pore distances of the mesoporous structures were obtained from the first Bragg peak, the *d*_(10)_ reflection. The lattice constant *a* was calculated by 2*d*_(10)_/(3)^1/2^. The pore diameter and the pore wall thickness were calculated from the peak intensities using a two-phase model with an analytical approach [36–37] or alternatively from an electron density reconstruction with the appropriate choice of phases for hexagonal structures [11,37–38].

For comparison, the pore size was obtained from the Barrett–Joyner–Halenda (BJH) model from the de- and adsorption branch of the isotherm and the pore wall thickness derived from the pore-to-pore distance from the SAXS measurements and subtraction of the corresponding pore diameter from the BJH model.

## Supporting Information

Supporting Information features a detailed description on the evaluation of SAXS data, as well as extensive measurement data on unmodified silica gels.

File 1 Evaluation of the SAXS data and measurements of unmodified gels.
