# Supplementary material for: Inorganic–organic hybrid materials through post-synthesis modification: Impact of the treatment with azides on the mesopore structure
Source: Beilstein J Nanotechnol. 2011 Aug 26;2:486–98. doi: 10.3762/bjnano.2.52 (PMC3190618; doi:10.3762/bjnano.2.52)
Supplement: File 1 — Evaluation of the SAXS data and measurements of unmodified gels. [file Beilstein_J_Nanotechnol-02-486-s001.pdf]

Supporting Information

for

Inorganic–organic hybrid materials through post-synthesis modification: Impact of the treatment with azides on the mesopore structure

Miriam Keppeler[1], Jürgen Holzbock[1], Johanna Akbarzadeh[2], Herwig Peterlik[2], Nicola Hüsing*[1,3]

Address: [1]Inorganic Chemistry I, Ulm University, Albert-Einstein Allee 11, D-89081 Ulm, Germany, [2]Dynamics of Condensed Systems, University of Vienna, Strudlhofgasse 4, A-1090 Vienna, Austria, and [3]Materials Chemistry, Paris-Lodron University Salzburg, Hellbrunner Str. 34, A-5020 Salzburg, Austria

Email: Nicola Hüsing* - nicola.huesing@sbg.ac.at

*Corresponding author

Evaluation of the SAXS data and measurements of unmodified gels

Evaluation of the SAXS data

Two phase model:

In this model we assume that the material is built up of two phases, one phase being hexagonally ordered pores embedded in a second phase, the silica matrix. Figure 1 shows schematically a hexagonal reciprocal lattice with the repeating unit distance $d_{10} = \frac{2\pi}{q_{\max}}$ and lattice constant $a = \frac{2}{\sqrt{3}} d_{10}$.

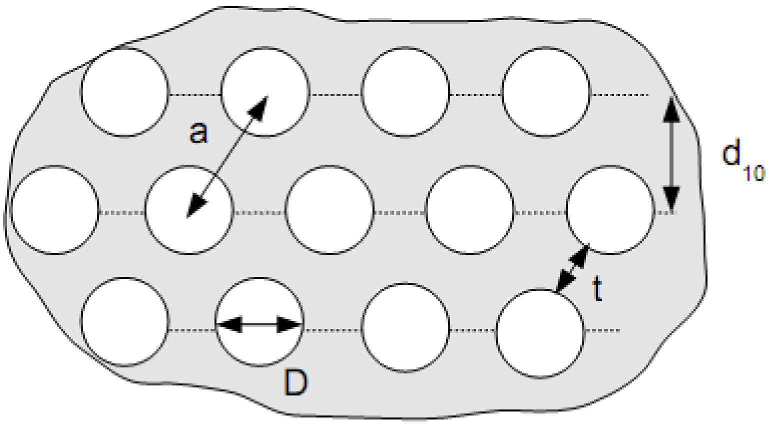

Figure S1: Schematic representation of a hexagonally organized pore system and the characteristic sizes

From a geometrical point of view, one can consider the lattice constant *a* to be:

$$a = D + t \tag{1}$$

with *D* being the mean pore-diameter and *t* the mean wall thickness between the pores. Variation of pore diameter and wall thickness leads to changes in the electron density distribution and thus to differences in the scattering intensities of the diffraction peaks.

According to Glatter and Kratky [1], the scattering intensity is proportional to the product of structure factor S(q) and the square of the scattering amplitude (form factor) P(q):

$$I(q) \propto A \cdot S(q) \cdot |P(q)|^2 \tag{2}$$

For the case of a 2-D hexagonal lattice with spherical pores, S(q) is described as the sum of Gaussian functions with maxima located at the reciprocal lattice points. The reciprocal lattice points are equal to the positions of the Bragg-reflections in the scattering curve

$q_{hk} = \frac{4\pi}{a\sqrt{3}} \sqrt{h^2 + hk + k^2}$ [2,3].

The form factor P(q) can be written as [4]:

$$|P(q)|^2 = \frac{(2\pi R)^2}{q} \left(\frac{J_1(qR)}{(qR)} \right)^2 \tag{3}$$

with R being the mean pore radius and J_1 the Bessel function of the first kind.
The scattering intensity (2) was then convoluted with the resolution function of the equipment described by a Lorentzian curve and fitted to the experimental values. Fitting parameters were the pore diameter and the wall thickness as described above.

Electron density reconstruction:

A different approach is the reconstruction of the electron densities from a Fourier series and by the appropriate choice of the phases [5,6]. This has been experimentally and theoretically used to model the electron density across the pore for modified and unmodified MCM-41 and SBA-15 materials [7,8]. For a centrosymmetric unit cell, the electron density can be written as a Fourier series of cosines:

$$\rho(\vec{r}) = \rho_{average} + \sum_{q} A_q \cos(\vec{q}\vec{r}) \tag{4}$$

With A_q being the Fourier coefficients, $\vec{q}$ the scattering vector of the reciprocal lattice and $\vec{r}$ a vector to the position in the unit cell.
The Fourier coefficients are determined from the peak intensities of the observed (h,k) reflections by

$$\left|A_{q_{hk}}\right| \propto \sqrt{\frac{I(q_{hk})LP(q_{hk})}{m}} \tag{5}$$

with $q_{hk} = \frac{4\pi}{a\sqrt{3}}\sqrt{h^2 + hk + k^2}$, $LP(q_{hk})$ being the Lorentz polarization-factor [9] and m the multiplicity of the respective reflection [5,6].

In order to obtain the integrated intensities I(q), the background scattering was subtracted from the data using a non-linear baseline. The remaining peaks were then fitted by a Gaussian function, from which the peak area was determined.

The next step in reconstructing the electron density is choosing the right phase. For centro-symmetric structures, this is equivalent to choosing the correct sign (+ or -) of the Fourier coefficients. The method, known as the "swelling method", was originally described by Stamatoff and Krimm [10] for lamellar phases (L_α) and was extended by Turner and Gruner [5] to hexagonal phases: One compares two phases with slightly differing radii and therefore slightly differing X-ray patterns. Then all possible permutations of signs for the A_q's are calculated and compared, the proper phase set will have the closest correspondence (for more details see [5,6]). In our case, we did not use a swelling of phases, but compared the samples before and after nucleophilic substitution, which led also to slightly differing X-ray patterns. Finally, one has to check if the resulting phase is physically consistent. For example, if the origin was placed in the centre of the pore cross section, the electron density should be a minimum at this point. Towards the pore wall, the electron density should increase and reach a maximum in the silica phase between the pores.

Nucleophilic substitution of chloro- by azido groups on the silica surface

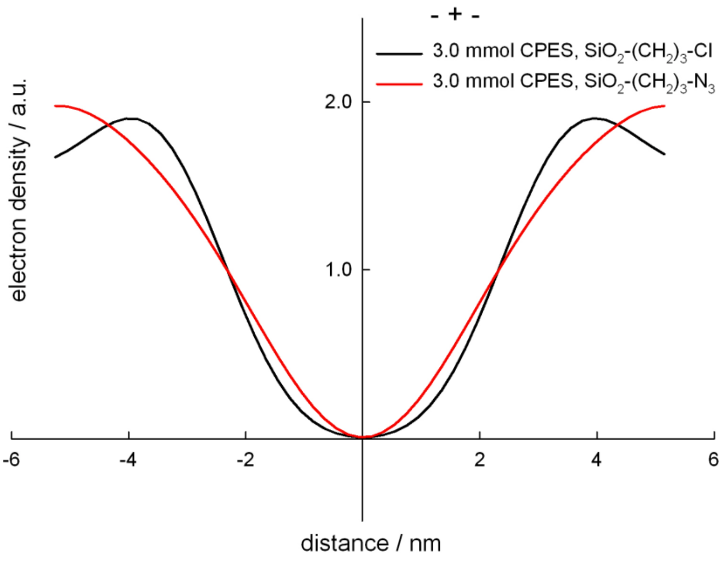

Figure S2: Electron-density reconstruction for modified silica gels (SiO_2-$(CH_2)_3$-Cl and SiO_2-$(CH_2)_3$-N_3) prepared from a silica-precursor solution containing 3.0 mmol CPES. The electron density of SiO_2-$(CH_2)_3$-Cl corresponds more to a sharp interface, whereas the broader distribution of SiO_2-$(CH_2)_3$-N_3 indicates a higher surface roughness due to the nucleophilic substitution.

Aging of unmodified silica gels in azide-containing media

Table S1: Structural characteristics of untreated silica, reference silica (solvent/60 °C) and azide-treated silica gels (solvent/NaN_3/60 °C) from nitrogen sorption analysis, solvents: TMU and H_2O

	S_{BET} [a] [m^2 g^{-1}]	C_{BET}	V_{max} [cm^3 g^{-1}]	$D_{BJH, Des}$ [b] [nm]	$D_{BJH, Ads}$ [c] [nm]
SiO_2	521	63.4	478.0	6.22	7.52
SiO_2/ TMU	866	59.0	825.6	6.74	9.15
SiO_2/TMU/NaN_3	722	47.3	915.5	7.99	11.73
SiO_2	532	55.7	496.4	6.19	9.41
SiO_2/H_2O	641	69.9	628.4	6.71	9.15
SiO_2/H_2O/NaN_3	515	47.9	814.9	8.03	11.66

[a]Calculated in the BET model. [b]Calculated from the desorption isotherm in the BJH model. [c]Calculated from the adsorption isotherm in the BJH model.

Table S2: Structural characteristics, as obtained from SAXS analyses, of untreated silica, reference silica (solvent/60 °C) and azide-treated silica gels (solvent/NaN_3/60 °C), solvents: TMU and H_2O

	$q_{(10)}$ [a] [nm^{-1}]	$d_{(10)}$ [a] [nm]	a [b] [nm]	t_{Des} [c] [nm]	t_{Ads} [d] [nm]	Mean pore diameter [e] ±0.2 [nm]	mean wall thickness [e] ±0.2 [nm]
SiO_2	0.57	10.95	12.64	6.21	4.91	8.59	4.11
SiO_2/ TMU	0.56	11.26	13.01	6.32	3.91	8.68	4.42
SiO_2/TMU/NaN_3	0.54	11.64	13.44	5.40	1.68	9.30	4.20
SiO_2	0.57	11.07	12.79	6.55	3.33	8.50	4.20
SiO_2/H_2O	0.58	10.85	12.53	5.72	3.28	8.55	4.05
SiO_2/H_2O/NaN_3	0.55	11.44	13.22	5.03	1.40	9.22	4.08

[a]Calculated from SAXS measurements, $q_{(10)} = (4\pi/\lambda)\sin\Theta$, $d_{(10)}$ calculated by the Bragg equation. [b]Lattice constant, calculated by $2d_{(10)}/(3)^{1/2}$. [c]Wall thickness, calculated by a – $D_{BJH, Des}$. [d]Wall thickness, calculated by a – $D_{BJH, Ads}$. [e]Mean pore diameter and wall thickness calculated from the peak intensities (SAXS) by a two-phase model with an analytical approach.

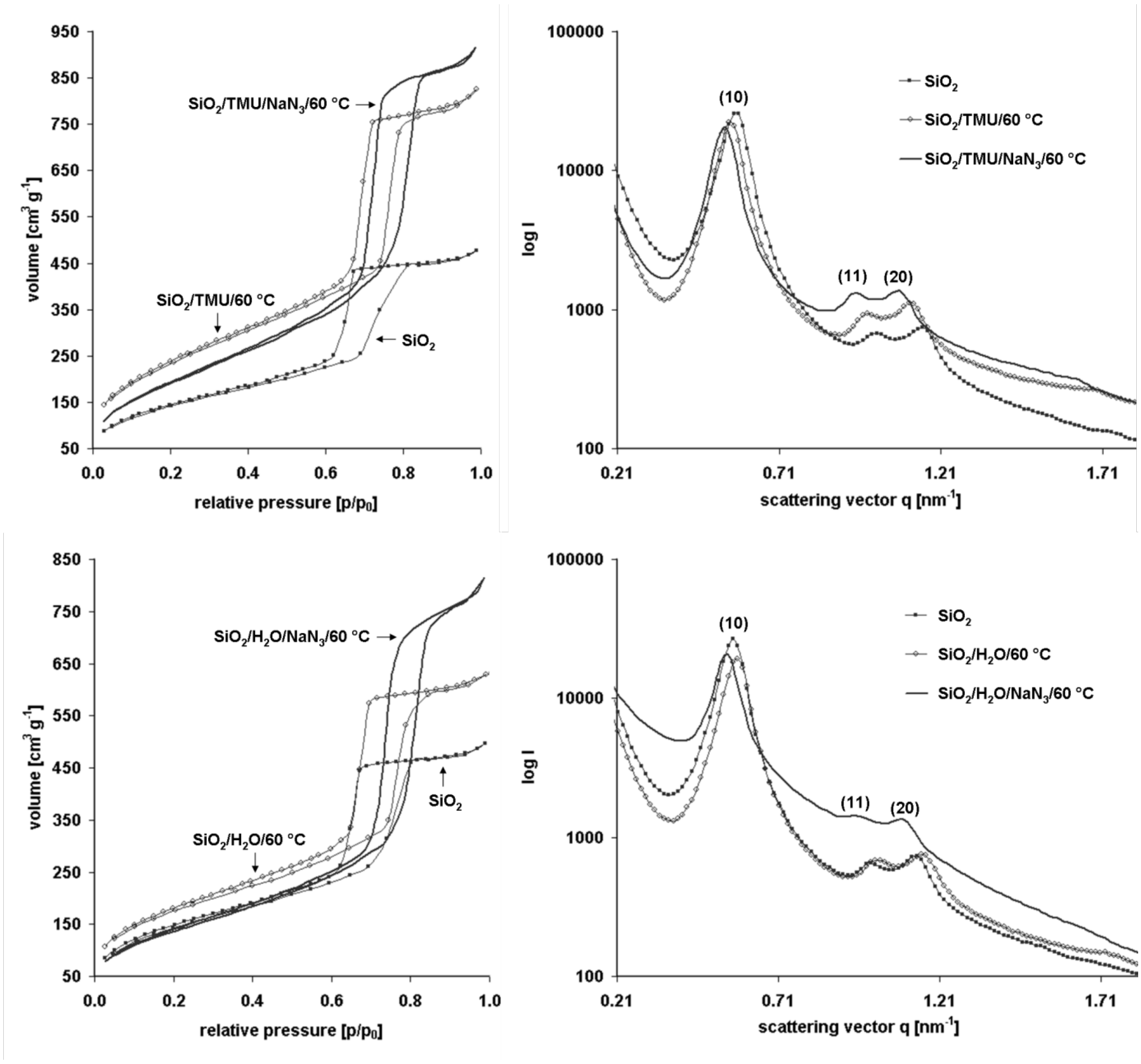

Figure S3: Nitrogen isotherms and SAXS patterns of untreated silica gels, reference silica gels (solvent/60 °C) and azide-treated silica gels (solvent/NaN_3/60 °C) in different solvents: TMU (top) and H_2O (bottom)

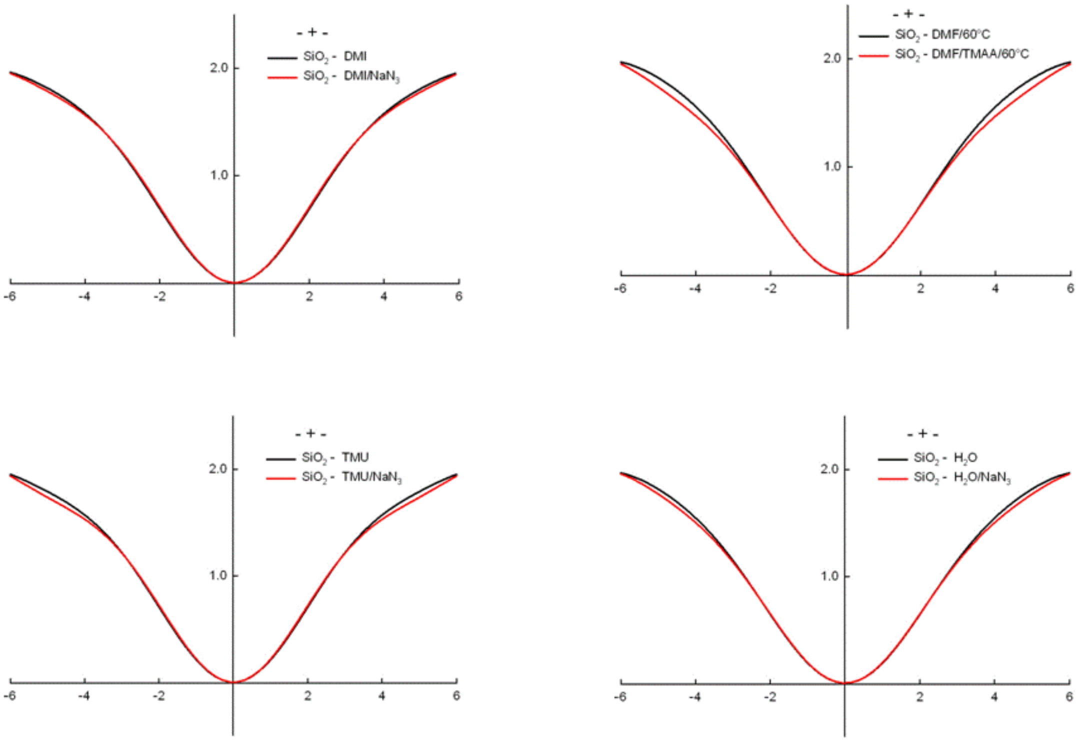

Figure S4: Electron-density reconstruction for unmodified silica gels exposed to DMI and DMI/NaN_3 (top left), TMU and TMU/NaN_3 (bottom left), H_2O and H_2O/NaN_3 (bottom right) and for gels exposed to DMF and DMF/TMAA (top right).

References

1. Glatter, O.; Kratky, O. *Small Angle X-Ray Scattering;* Academia Press: London, 1982.
2. Jähnert, S.; Müter, D.; Prass, J.; Zickler, G. A.; Paris, O.; Findenegg, G. H. *J. Phys. Chem. C* **2009,** *113,* 15201–15210. doi:10.1021/jp8100392
3. Zickler, G. A.; Jähnert, S.; Wagermaier, W.; Funari, S. S.; Findenegg, G. H.; Paris, O. *Phys. Rev. B* **2006,** *73,* 184109. doi:10.1103/PhysRevB.73.184109
4. Guinier, A.; Fournet, G. *Small Angle Scattering of X-Rays;* Wiley: New York, 1955.
5. Turner, D. C.; Gruner, S. M. *Biochem.* **1992,** *31,* 1340–1355. doi:10.1021/bi00120a009
6. Harper, P. E.; Mannock, D. A.; Lewis, R. N. A. H.; McElhaney, R. N.; Gruner, S. M. *Biophys. J.* **2001,** *81,* 2693–2706. doi:10.1016/S0006-3495(01)75912-7
7. Flodström, K.; Teixrira, C.V.; Amenitsch, H.; Alfredsson, V.; Lindén, M. *Langmuir* **2004,** *20,* 4885–4891. doi:10.1021/la049637c
8. Beurroies, I.; Agren, P.; Buchel, G.; Rosenholm, J.B.; Amenitsch, H.; Denoyel, R.; Lindén, M. *J. Phys. Chem.* **2006**, *110*, 16254–16260.
9. Warren, B.E. *X-Ray Diffraction;* Dover Publications: New York, 1990.
10. Stamatoff, J. B.; Krimm, S. *Biophys. J.* **1976,** *16,* 503–516. doi:10.1016/S0006-3495(76)85705-0
